# The clinical effectiveness and cost effectiveness of clozapine for inpatients with severe borderline personality disorder (CALMED study): a randomised placebo-controlled trial

**DOI:** 10.1177/20451253221090832

**Published:** 2022-04-29

**Authors:** Mike J. Crawford, Verity C. Leeson, Rachel Evans, Barbara Barrett, Aisling McQuaid, Jack Cheshire, Rahil Sanatinia, Gary Lamph, Piyal Sen, Katina Anagnostakis, Louise Millard, Inti Qurashi, Fintan Larkin, Nusrat Husain, Paul Moran, Thomas R.E. Barnes, Carol Paton, Zoe Hoare, Marco Picchioni, Simon Gibbon

**Affiliations:** Division of Psychiatry, Imperial College London, The Commonwealth Building, The Hammersmith Hospital, Du Cane Road, London W12 0NN, UK; Division of Psychiatry, Imperial College London, London, UK; North Wales Organisation for Randomised Trials in Health, Bangor University, Bangor, UK; King’s Health Economics, King’s College London, London, UK; Division of Psychiatry, Imperial College London, London, UK; Department of Forensic Psychiatry, Nottinghamshire Healthcare NHS Foundation Trust, Nottingham, UK; Division of Psychiatry, Imperial College London, London, UK; School of Nursing, University of Central Lancashire, Preston, UK; Department of Forensic Psychiatry, Elysium Healthcare, Milton Keynes, UK; St Andrew’s Academic Centre, St Andrew’s Healthcare, Northampton, UK; St Andrew’s Academic Centre, St Andrew’s Healthcare, Northampton, UK; Ashworth Hospital, Mersey Care NHS Foundation Trust, Liverpool, UK; Acute Mental Health Services, West London NHS Trust, London, UK; Division of Psychology & Mental Health, University of Manchester, Manchester, UK; Centre for Academic Mental Health, University of Bristol, Bristol, UK; Division of Psychiatry, Imperial College London, London, UK; Division of Psychiatry, Imperial College London, London, UK; North Wales Organisation for Randomised Trials in Health, Bangor University, Bangor, UK; Department of Forensic and Neurodevelopmental Science, Kings College London, London, UK; Department of Forensic Psychiatry, Nottinghamshire Healthcare NHS Foundation Trust, Nottingham, UK

**Keywords:** Borderline personality disorder, Clinical Trial, clozapine

## Abstract

**Background:**

Data from case series suggest that clozapine may benefit inpatients with borderline personality disorder (BPD), but randomised trials have not been conducted.

**Methods:**

Multicentre, double-blind, placebo-controlled trial. We aimed to recruit 222 inpatients with severe BPD aged 18 or over, who had failed to respond to other antipsychotic medications. We randomly allocated participants on a 1:1 ratio to receive up to 400 mg of clozapine per day or an inert placebo using a remote web-based randomisation service. The primary outcome was total score on the Zanarini Rating scale for Borderline Personality Disorder (ZAN-BPD) at 6 months. Secondary outcomes included self-harm, aggression, resource use and costs, side effects and adverse events. We used a modified intention to treat analysis (mITT) restricted to those who took one or more dose of trial medication, using a general linear model fitted at 6 months adjusted for baseline score, allocation group and site.

**Results:**

The study closed early due to poor recruitment and the impact of the COVID-19 pandemic. Of 29 study participants, 24 (83%) were followed up at 6 months, of whom 21 (72%) were included in the mITT analysis. At 6 months, 11 (73%) participants assigned to clozapine and 6 (43%) of those assigned to placebo were still taking trial medication. Adjusted difference in mean total ZAN-BPD score at 6 months was -3.86 (95% Confidence Intervals = -10.04 to 2.32). There were 14 serious adverse events; 6 in the clozapine arm and 8 in the placebo arm of the trial. There was little difference in the cost of care between groups.

**Interpretation:**

We recruited insufficient participants to test the primary hypothesis. The study findings highlight problems in conducting placebo-controlled trials of clozapine and in using clozapine for people with BPD, outside specialist inpatient mental health units.

**Trial registration:**

ISRCTN18352058. https://doi.org/10.1186/ISRCTN18352058

## Introduction

Borderline personality disorder (BPD) is a common mental health condition that is characterised by instability in emotions, identity, and interpersonal relationships.^1^ Some people with severe BPD have very high levels of contact with inpatient mental health services.^2^ It is estimated that more than a fifth of inpatients on general adult wards,^3,4^ more than 40% of women on Psychiatric Intensive Care Units,^5^ and more than 60% of women treated on medium secure units have BPD.^6^

While psychological treatments improve the mental health of people with BPD,^7^ people with the most severe problems are less likely to engage in them.^8,9^ No medication is licenced for the treatment of BPD, but despite this, people with severe BPD are often prescribed multiple medications.^10,11^ Clozapine is used to treat people with schizophrenia who have not responded to other antipsychotic medications.^12^ As well as improving the mental health of people with psychosis, clozapine appears to reduce the incidence of aggressive and impulsive behaviour^13^ and it is licenced in the United States for reducing the risk of recurrent suicidal behaviour among people with schizophrenia.^14^

Open label studies of clozapine for people with borderline personality disorder have reported improved mental health, reductions in aggressive and self-harming behaviour, and lower costs of care.^13,15,16^ In an observational study of 102 Danish patients with BPD who had ever been prescribed clozapine, levels of self-harm were found to be lower when people took this medication compared to when they did not.^17^ Qualitative data collected from women with borderline personality disorder treated with clozapine on secure wards also highlight potential benefit, with many describing marked improvements in their mental health and relationships with others.^18^ While it is possible that clozapine improves the mental health of people with borderline personality disorder, it has serious side effects including weight gain and associated metabolic complications, constipation, seizures and potentially fatal agranulocytosis, pneumonia, myocarditis and paralytic ileus.^19–21^ People prescribed clozapine are therefore required to have regular checks on their physical health including weekly blood tests for the first 18 weeks of treatment. Previous placebo-controlled trials of medication for people with borderline personality disorder have shown clear evidence of improved outcomes for those prescribed placebos.^22^ We do not know to what extent the benefits of clozapine seen in open-label studies can be explained by non-specific factors including instillation of hope and the structured care that people taking clozapine receive through additional monitoring of their physical health.

We therefore designed a placebo-controlled trial to test the clinical effectiveness and cost-effectiveness of clozapine for inpatients with borderline personality disorder. We followed up participants 3 and 6 months after randomisation to examine the clinical effects, costs and safety of this treatment.

## Methods

### Study design

The CALMED (Clozapine Assessing Long-term Medication in Emotionally unstable personality Disorder) study is a two-arm, parallel group, blinded, randomised trial of clozapine versus placebo for inpatients with a diagnosis of borderline personality disorder. We recruited inpatients aged 18 years or over who had severe borderline personality disorder and were being treated by one of seven NHS and independent sector organisations in England. Potential participants had to meet *DSM*-IV diagnostic criteria for borderline personality disorder using the Structured Clinical Interview for Axis II Personality Disorders.^23^ Severity of personality disorder was based on service use and risk. To take part in the study potential participants had to have been an inpatient on a mental health ward for more than 28 days in the last 12 months, or to have had two or more admissions to hospital/periods of care provided by Home Treatment over the last 12 months, plus a lifetime history of two or more incidents of harm to self or others which resulted in permanent damage, or would have done so had services not intervened. Potential participants had to have not shown an adequate clinical response despite taking antipsychotic medication other than clozapine for at least 3 months. They also needed to have their current body weight and blood glucose level recorded in their clinical records and have a satisfactory pre-treatment full blood count (white blood cell count > = 3.5 x10^9^/L and absolute neutrophil count > = 2.0 x10^9^/L). We excluded those who had a current coexisting clinical diagnosis of schizophrenia, or bipolar I disorder, and those who had been prescribed clozapine within the last 2 weeks, as well as those women known to be pregnant, trying to conceive, breastfeeding, or who were of childbearing potential but not using a highly effective birth control. We also excluded any potential participant who was due to be discharged within the following 2 weeks unless arrangements for necessary monitoring of physical health as an outpatient could be made. We excluded those unable to speak sufficient English to complete the baseline assessment, those unable to undergo regular blood tests, those with contraindications for clozapine and those unwilling or unable to provide written informed consent to take part in the study.

The study was approved by the Wales Research Ethics Committee 1 Research Ethics Committee (Ref: 217828). The Medicines and Healthcare Products Regulatory Agency (MHRA) gave Clinical Trial Authorisation and the Research and Development departments of the participating provider organisations approved the trial prior to the start of recruitment. The participants provided written informed consent. This trial was prospectively registered at ISRCTN with the ID #18352058: https://doi.org/10.1186/ISRCTN18352058

### Randomisation and masking

Eligible participants were asked to complete the Psychosis Screening Questionnaire^24^ and the International Personality Disorder Examination (IPDE) Screening Questionnaire^25^ and all were enrolled with the Clozaril Patient Monitoring Service. We used this service to ensure that no study participant could be administered study medication if they had a significantly reduced white blood cell count during the previous 7 days during the first 18 weeks of treatment and for 14 days thereafter (white blood cell count  < 3.0 x10^9^/L and absolute neutrophil count  < 1.5 x10^9^/L).

We then randomly allocated participants on a 1:1 ratio to either clozapine or placebo via a secure and automated randomisation service operated by NWORTH, University of Bangor. We used a sequentially randomised, dynamic adaptive algorithm, stratified by centre, ward type and gender.^26^ A trial prescription form was then completed by the responsible clinician and sent to a local pharmacy for dispensing to the ward where the participant was being treated.

All study researchers, aside from the trial manager, remained masked to allocation status until after an initial discussion of the trial findings was completed. We asked participants and researchers to guess allocation status after they had collected 6-month follow-up data.

### Interventions

All those taking part in the study continued to receive usual treatment including inpatient psychosocial care prior to transfer to another ward, or discharge to the community contingent upon their mental health and risk assessment. We imposed no restrictions on the use of other treatments, except that the participants who remained in the trial could not be prescribed clozapine. Those in the active arm of the trial were prescribed up to 300 mg of clozapine titrated over a 15-day period. The dose could then be increased to 400 mg of clozapine daily, depending on clinical response, patient preference and side effects. Those randomised to control treatment were prescribed an inert placebo capsules that were identical in appearance but contained a microcrystalline cellulose backfill.

### Outcomes

The primary outcome was total score on the Zanarini rating scale for Borderline Personality Disorder (ZAN-BPD) at 6 months.^27^ The ZAN-BPD is a widely used outcome in trials for BPD and provides a reliable and valid assessment of core features of the condition and is sensitive to change.^28,29^ A higher score on the measure indicates poorer mental health.

The secondary outcomes were;

i. Total score on the Zanarini rating scale for Borderline Personality Disorder at 3 months.
ii. General mental health using the Brief Psychiatric Rating Scale (BPRS) at 3 and 6 months.^30^ The BPRS has been used in previous open-label studies of clozapine in people with borderline personality disorder.^13,16^
iii. Incidence and severity of suicidal behaviour using the Acts of Deliberate Self-Harm Inventory.^31^
iv. Level of aggressive behaviour using the Modified Overt Aggression Scale (MOAS).^32^
v. Health related quality of life using the EuroQuol-5 Dimensions (EQ-5D-5 L).^33^
vi. Side effects of medication using the Antipsychotic Non-Neurological Side Effects Scale (ANNSERS)^34^ and motor and extrapyramidal side effects using the Extrapyramidal Side Effects Scale.^35^
vii. Incidence of withdrawal of trial medication due to adverse effects.
viii. Medication adherence at 3 and 6 months using the Brief Adherence Rating Scale (BARS).^36^
ix. Indication of the severity of personality problem collected at baseline and 6 months using the self-report Standardised Assessment of Severity of Personality Disorder (SAS-PD).^37^
x. Resource use collected using a modified version of the Adult Service Use Schedule.^38^ This includes detailed information about length of inpatient treatment and type of ward, contacts with community mental health services and emergency medical services, and the type and dose of psychotropic medication that people were prescribed.

### Statistical analysis

Using PASS software (NCSS, Kaysville, Utah, USA) we calculated that we would need to analyse data from 166 participants (83 receiving clozapine and 83 receiving placebo) to have 90% power to detect a four-point clinically important difference in ZAN-BPD score at 6 months, with an assumed standard deviation of 7.89 and a significance level of 0.05 using a two-sided two sample t-test. To take account of 25% loss to follow-up we planned to recruit 222 subjects.

The analysis of both the primary and secondary outcomes used a linear mixed model adjusted for baseline score along with site as a random effect. For the primary analysis we used a modified intention to treat analysis (mITT), using data from all randomised participants who took at least one dose of trial medication. Where the number of missing items on an outcome measure was 20% or less, we substituted the individual’s mean score for the remaining items on the scale. If there were more than 20% missing items on the scale, the outcome measure was not calculated for the participant at that time point and multiple imputation methods were used where appropriate.

We undertook secondary exploratory sensitivity analyses. A per protocol analysis was conducted in which we analysed data from a subset of participants who took trial medication until the first follow-up interview at three months, in order to explore the effect of clozapine among people who took it. We undertook sensitivity analysis of patient-reported lethargy, derived from the ANNSERS,^34^ on the primary outcome by including this as a factor in the model. We also conducted a sensitivity analysis of trial arm perception of both the participant and the researcher; the primary analysis model was run including allocation perception to assess any impacts on the scores. Finally, we examined differences in the primary outcome in different treatment settings: general adult wards, low, medium and high secure units. All statistical tests were two-sided using a 5% significance level and we calculated 95% confidence intervals for estimated effect sizes.

The economic analysis took a health and personal social services perspective, which is relevant to UK decision makers and to this patient group.^39^ We collected service use data using a modified version of the Adult Service Use Schedule (AD-SUS), which was based on our previous research in this group and was piloted and adapted in collaboration with clinical and service user members of the research team. Total costs were calculated by matching each service use item with a relevant unit cost, which were sourced from routine UK sources.^40,41^ Primary economic analysis was at 6-month follow-up. Differences in service use were explored descriptively and differences in costs were calculated using standard t-tests adjusted for baseline costs and with bootstrapped confidence intervals, as recommended for cost data.^42^

## Results

**Figure 1.**
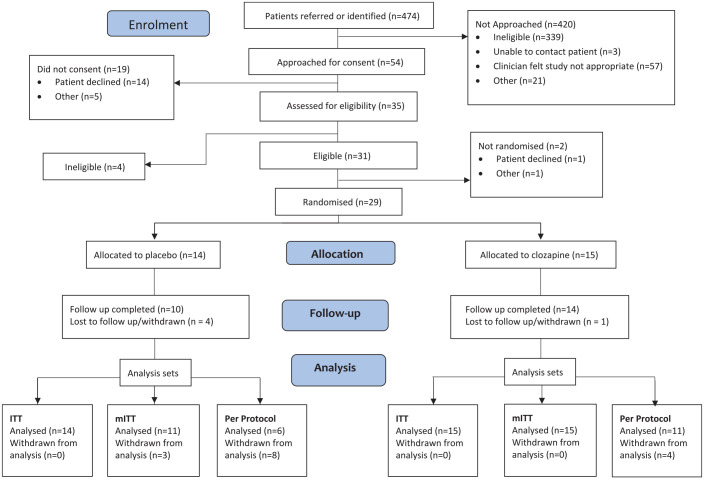
CONSORT 2010 flow diagram.

Recruitment took place from September 2019 to March 2020 and was then suspended due to the COVID-19 pandemic. We attempted to restart recruitment in autumn 2020, but researchers were no longer permitted to visit participating sites due to infection control policies. Continuing pressures on clinical staff resulting from the pandemic and concerns about the off-label use of clozapine during the crisis that further limited recruitment, which subsequently closed in February 2021. We assessed the records of 474 inpatients for eligibility (see Figure 1). Of these, 339 (93%) had a diagnosis of schizophrenia, bipolar I disorder or a related psychosis and were excluded. Of the remaining 135, clinicians judged 57 (42%) to be unsuitable for the study. Of 54 potential participants approached for consent and further assessment, 35 consented, 31 were eligible and 29 were randomised. The main reason why eligible participants declined to take part in the study was that they were unwilling to take clozapine or placebo. Fifteen participants were randomised to clozapine and 14 to placebo. Table 1 presents a summary of baseline characteristics of the two groups.

**Table 1.** Summary characteristics of study participants by intervention group.

Variable	Overall *N* = 29	Placebo *N* = 14	Clozapine *N* = 15
**Age:** mean (SD)	30 (9.74)	33 (11.25)	28 (7.54)
**Self-identified gender:** *N* (%)			
Male	7 (24%)	3 (21%)	4 (27%)
Female	22 (76%)	11 (79%)	11 (73%)
**Ethnicity:** *N* (%)			
White – British	25 (86%)	11 (79%)	14 (93%)
White – Irish	1 (3%)	1 (7%)	0 (0%)
White – Other	1 (3%)	1 (7%)	0 (0%)
Other	2 (7%)	1 (7%)	1 (7%)
**Ward type:** *N* (%)			
General adult	9 (31%)	4 (29%)	5 (33%)
Low secure	5 (17%)	3 (21%)	2 (13%)
Medium secure	14 (48%)	6 (43%)	8 (53%)
High secure	1 (4%)	1 (7%)	0 (0%)
**Mental Health Act status at baseline:** *N* (%)			
Voluntary inpatient	6 (21%)	3 (21%)	3 (20%)
Section 2	1 (3%)	0 (0%)	1 (7%)
Section 3	14 (48%)	7 (50%)	7 (47%)
Section 37/38	4 (14%)	2 (14%)	2 (13%)
Section 41	4 (14%)	2 (14%)	2 (13%)
Missing	2 (7%)	1 (7%)	1 (7%)
**Attempted suicide over last six months:** *N* (%)			
Yes	21 (72%)	10 (71%)	11 (73%)
No	8 (28%)	4 (29%)	4 (27%)
**Self-harm over last six months:** *N* (%)			
Yes	24 (83%)	11 (79%)	13 (87%)
No	5 (17%)	3 (21%)	2 (13%)
**Zanarini rating scale for Borderline Personality Disorder: mean (SD)**	19.7 (7.71)	18.5 (8.31)	20.9 (7.21)
**Antipsychotic Non-Neurological Side Effects Scale:** mean (SD)	14.9 (12.56)	14.6 (15.91)	15.1 (8.63)
**Brief Psychiatric Rating Scale:** mean (SD)	53.6 (12.56)	55.1 (14.57)	52.3 (10.69)
**Modified Overt-Aggression Scale:** mean (SD)	29.5 (23.15)	26.6 (25.87)	32.3 (20.81)
**Standardised Assessment of Severity of Personality Disorder:** mean (SD)	14.2 (3.47)	13.9 (3.44)	14.6 (3.58)
**European Quality of Life-5 Dimensions -3 L Utility score:** mean (SD)	0.430 (0.358)	0.474 (0.379)	0.389 (0.345)

Three-quarters of the study sample were female and the mean age was 30 years (range = 18 to 53). Most of the sample (*n* = 23, 79.3%) were being treated on a compulsory basis and half (*n* = 14, 48%) were recruited from medium secure units. In addition to meeting *DSM*-IV criteria for borderline personality disorder, self-report data from the IPDE, which was completed by 27 participants, indicated that most participants met criteria for probable avoidant (*n*  = 27, 93%), schizotypal (*n* = 26, 90%), compulsive (*n* = 22, 76%), dependent (*n* = 22, 76%), schizoid (*n* = 21, 72%), histrionic (*n* = 20, 69%), and paranoid 19 (66%) personality disorder. At baseline, 26 participants (90%) had a score of 10 or more on the SASPD, the threshold for moderate/ severe personality disorder (12 out of 14 (86%) in the placebo arm and 14 out of 15 (93%) in the clozapine arm of the trial).

Table 2 summarises the data on adherence with trial medication during the study. All 15 in the clozapine arm of the trial started trial medication, as did 11 (79%) of 14 in the placebo arm of the trial. One participant randomised to placebo withdrew their consent prior to taking their first dose of trial medication. Two were withdrawn from the study because they were discharged earlier than planned and before arrangements could not be made for monitoring their physical health in the community. At 6-month follow-up, 11 (73%) of 15 people in the clozapine arm of the trial and six (43%) of 14 participants in the placebo arm were still taking trial medication. Reasons for stopping trial medication were: withdrawal of patient consent (*n* = 4), non-compliance (*n* = 2), adverse event (*n* = 1), physician decision (*n* = 1) and problems encountered with blood monitoring when a patient was discharged to a community setting. Levels of adherence among those who continued to take trial medication were very high, with a mean percentage score on the Brief Adherence Rating Scale of 98.2 (SD = 2.51) at 3 months and 99.0 (SD = 1.56) at 6 months.

**Table 2.** Adherence with trial medication.

Discontinued study medication	3 months follow-up			6 months follow-up		
	**Overall** *N* **= 29**	Placebo *N* = 14	Clozapine *N* = 15	**Overall** *N* **= 29**	Placebo *N* = 14	Clozapine *N* = 15
Yes	**13 (45%)**	9 (64%)	4 (27%)	**14 (48%)**	11 (79%)	4 (27%)
No	**16 (55%)**	5 (36%)	11 (73%)	**15 (52%)**	4 (29%)	11 (73%)

Among 12 participants in the clozapine arm of the trial who guessed their allocation status, 8 thought they had been taking clozapine and 4 did not know what they were taking. Of six participants in the placebo arm of the trial who guessed their allocation status, five believed they were taking a placebo and one stated that they did not know. Researchers correctly guessed that 11 of 12 participants in the active arm of the trial were allocated to clozapine and five of the six participants in the control arm of the trial were allocated to placebo.

**Figure 2.**
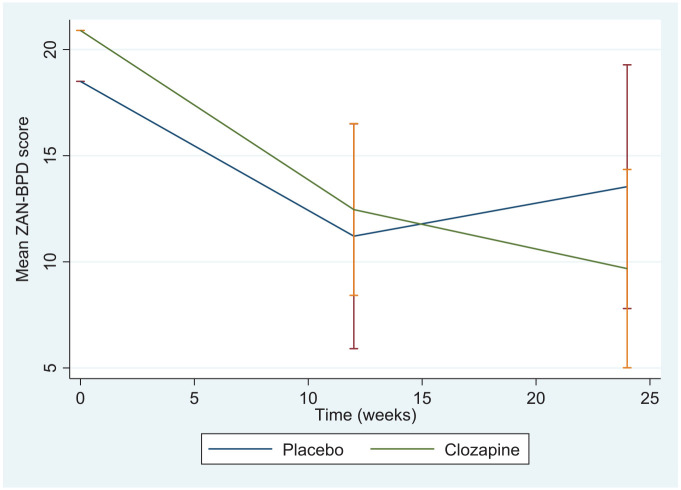
Change in adjusted mean total ZAN-BPD score at 3 and 6 months (error bards represent 95% Confidence Intervals of mean scores).

There were no statistically significant differences in study outcomes between those in the clozapine and placebo arm of the trial (see Table 3 and Figure 2). ZAN-BPD was 9.68 (SD = 2.38) in the clozapine arm and 13.54 (SD = 2.92) in the placebo arm (adjusted difference in means = -3.86, 95%, C.I = -10.04 to 2.32, *p* = 0.22) at 6 months. For the secondary per protocol analysis, the adjusted mean ZAN-BPD was 7.61 (SD = 2.30) in the clozapine arm and 12.77 (SD = 3.16) in the placebo arm (adjusted difference in means = -5.15, 95%, C.I = -12.06 to 1.75, *p* = 0.14) at 6 months. When lethargy was added to the model, the adjusted difference in mean ZAN-BPD score = -8.54, 95%, C.I = -13.31 to -3.75, *p* < 0.01). Among participants recruited from medium secure units, ZAN-BPD scores changed from 19.1 at baseline to 10.1 at 6 months among those in the clozapine arm, and from 19.2 (SD = 7.7) to 14.5 (SD = 7.6) among those in the placebo arm of the trial.

**Table 3.** Trial outcomes at 3 and 6 months.

OUTCOME	data set	Time 3point (months)	N	^a^Maximum likelihood estimates				Adjusted values	
				Adjusted difference	SE	95% CI		Placebo	Clozapine
						Lower	Upper	Mean (SE)	Mean (SE)
**ZAN-BPD**	mITT	6	26	−3.86	3.13	−10.04	2.32	13.54 (2.92)	9.68 (2.38)
**ZAN-BPD**	mITT	3	26	1.26	3.12	−4.88	7.39	11.21 (2.69)	12.46 (2.06)
**ZAN-BPD**	Per protocol	6	16	−5.15	3.52	−12.06	1.75	12.77 (3.16)	7.61 (2.30)
**ZAN-BPD (lethargy)**	mITT	6	21	−8.54	2.44	−13.31	−3.75	16.60 (2.83)	8.07 (246)
**ZAN-BPD (participant allocation perception)**	mITT	6	18	−13.62	6.21	−25.80	−1.45	18.41 (4.40)	4.79 (2.51)
**ZAN-BPD (participant Researcher perception)**	mITT	6	18	−4.87	6.92	−18.43	8.70	11.84 (5.06)	6.97 (3.06)
**ANNSERS**	mITT	3	25	4.34	4.14	−3.82	12.49	10.88 (3.08)	15.22 (2.41)
**ANNSERS**	mITT	6	25	3.06	4.00	−4.79	10.91	11.92 (2.98)	14.99 (2.43)
**BPRS**	mITT	3	26	3. 50	3.52	−3.44	10.44	38.53 (3.05)	42.03 (2.37)
**BPRS**	mITT	6	26	−4.65	3.66	−11.86	2.56	40.52 (3.48)	35.87 (2.70)
**MOAS**	mITT	3	26	7.35	4.41	−1.31	16.01	6.63 (3.44)	13.98 (2.80)
**MOAS**	mITT	6	26	0.45	5.95	−11.24	12.14	9.23 (5.11)	9.69 (4.18)
**SASPD**	mITT	6	26	−1.56	1.62	−4.73	1.61	12.12 (1.22)	10.56 (1.04)

ANNSERS, Antipsychotic Non-Neurological Side Effects Scale; BPRS, Brief Psychiatric Rating Scale; MOAS, Modified Overt-Aggression Scale; SASPD, Standardised Assessment of Severity of Personality Disorder; ZAN-BPD, Zanarini rating scale for Borderline Personality Disorder.

a Statistics presented are for clozapine vs the placebo group.

Regarding patient safety, 68 adverse events and 49 adverse reactions were reported among participants in the clozapine arm of the trial and 31 adverse events and 8 adverse reactions were reported among those in the placebo arm of the trial. Adverse events were predominantly mental health-related, including deterioration in mental health and self-harm. The main adverse reactions were gastrointestinal (including constipation), sedation and cardiac (including tachycardia). There were also 14 serious adverse events; six among those in the clozapine arm of the trial and eight among those in the placebo arm of the trial.

The use of services over the 6-month follow-up period is summarised in Table 4 and shows a similar pattern in the clozapine and placebo arms of the trial with substantial use of secondary health care, particularly inpatient stays. Total costs over 6 months were £68,876 in the placebo group and £72,599 in the clozapine group; the £3,723 difference in cost was not statistically significant and there were no significant differences in the utility score of the EQ-5D.

**Table 4.** Use of services, and utility scores over the 6-month follow-up period.

Component of care	Placebo (*n* = 10)			Clozapine (*n* = 14)		
	Mean	(SD)	% using services	Mean	(SD)	% using service
Hostel accommodation	0.00	(0.00)	0%	3.00	(7.04)	21%
Hostel accommodation - 24 h staff (months)	0.00	(0.00)	0%	0.00	(0.00)	0%
Hotel/*B*&B (months)	0.00	(0.00)	0%	0.00	(0.00)	0%
Inpatient stay (days)	127.00	(83.92)	90%	133.64	(75.14)	86%
Observation–intermittent (days)	68.00	(80.28)	50%	57.07	(73.05)	57%
Observation–in eye line (days)	3.10	(9.45)	20%	8.00	(9.31)	29%
Observation–at arm’s length (days)	0.00	(0.00)	0%	0.14	(0.53)	7%
Outpatient appointments (number)	2.30	(4.50)	40%	2.71	(3.36)	64%
Accident and emergency (attendances)	0.90	(1.37)	40%	1.79	(2.49)	57%
Community care (contacts)	8.10	(10.96)	40%	7.25	(10.75)	36%
GP (contacts)	7.00	(2.40)	100%	6.07	(2.09)	100%
Practice nurse (contacts)	0.10	(0.32)	10%	0.21	(0.58)	14%
Psychiatrist (contacts)	1.70	(5.03)	20%	1.43	(3.74)	29%
Psychologist (contacts)	0.70	(1.89)	20%	0.05	(1.16)	21%
Community psychiatric nurse (contacts)	6.70	(13.30)	40%	0.07	(0.03)	7%
Occupational therapist (number)	1.20	(3.79)	10%	1.21	(4.00)	14%
Social worker (contacts)	0.03	(0.95)	10%	0.57	(1.40)	21%
Medication (number)	9.30	(3.33)	100%	8.07	(4.60)	100%
EuroQuol-5 Dimensions-5 L utility score	0.50	(0.46)	–	0.61	(0.42)	–

We obtained data on 25 (86.2%) participants after they completed the 6-month follow-up interview to see if they were prescribed clozapine after the trial period. Of the 15 participants in the clozapine arm of the trial, 9 (60%) continued to be prescribed it. This included all eight of those in the clozapine arm who were being treated on inpatient units and one patient who had been treated on a general adult ward. Among 10 participants in the placebo arm of the trial, four were subsequently prescribed clozapine; all four participants were being treated as inpatients on specialist wards.

## Discussion

The CALMED study is the first randomised trial of clozapine for people with borderline personality disorder. Problems with recruitment, which were greatly compounded by the COVID-19 pandemic, meant that we fell well short of the sample size required for a fully powered study. Among the 26 participants who took at least one dose of trial medication and completed a 6-month follow-up assessment, there was a numerically lower ZAN-BPD score in the clozapine arm of the trial compared to those in the placebo arm, but this was not statistically significant (difference in adjusted means = -3.86, 95%, C.I = -10.04 to 2.32, *p* = 0.22). This finding is consistent with the improved outcomes associated with clozapine treatment reported in open-label studies.^13,15,16^ However, the magnitude of the difference between arms was small relative to the size of the placebo response; during the 6-month follow-up period, the general mental health of study participants in both arms of the study improved, and levels of aggression and self-harm halved among those in both the clozapine and the placebo arm of the trial. There were no major tolerability issues identified with clozapine, but again this finding must be interpreted in the context of our small sample size. Fourteen serious adverse events were reported during the trial (six in the clozapine arm and eight in the placebo arm of the trial). None of the 15 participants in the clozapine arm of the trial experienced sustained falls in white blood cell count nor experienced a serious adverse event that related to trial medication. However, one participant experienced a number of non-serious adverse events (hypersalivation, fatigue and constipation) that resulted in them stopping taking clozapine.

While we were only able to collect follow-up data from six people who took placebo, their improved mental health and the reductions in levels of aggression and self-harming behaviour, suggest that at least some of the benefits of clozapine reported in case-series may result from non-specific factors such as the instillation of hope, increased monitoring of physical health and regression to the mean.^43,44^

This trial has several important limitations, notably the small sample size. Recruitment was challenging in the first 6 months of the study, during which we recruited 25 participants (46% of the target during this period).^45^ The main reason for the low rate of recruitment was the reluctance of clinicians to refer people to the study. Clinicians on inpatient units often had strong views about the use of clozapine for people with BPD. Clinicians working on general adult wards usually had very little experience of prescribing clozapine to people with BPD. They reported concerns about the high side effect burden of clozapine and were worried that emotional instability and inconsistent contact with services would make it difficult or impossible to ensure that patients would get the regular physical health monitoring they required. In contrast, clinicians working on specialist wards reported having already seen the benefits of clozapine for people with severe BPD and were reluctant to involve their high-risk patients in a trial that could involve them being prescribed a placebo. Some patients on specialist inpatient wards shared these concerns. Having spoken to other patients on the ward who felt they had benefitted from taking clozapine, they decided that they did not want to take part in this trial. The wards where recruitment was most successful were in the independent sector, where clinicians were used to prescribing clozapine, but were also concerned about the potential for negative effects and were keen to improve the evidence base for pharmacotherapy for inpatients with borderline personality disorder. Recruitment on general adult wards was also limited because few patients with BPD were on the ward long enough to receive the regular physical health checks that are required when people are first prescribed this medication.^45^

Another limitation of the study was adherence to trial medication. Only 15 (52%) of our 29 study participants were taking trial medication at six months. Three people did not take a first dose of study medication. Among the 26 that did take at least one dose, 6 (55%) of those taking placebo and 4 (27%) of those in the clozapine arm of the trial were no longer taking it 6 months later. The reasons why people did not start trial medication or stopped it before the end of the follow-up period varied. In some instances, patients withdrew consent. In other instances, patients who had started to take trial medication were discharged from hospital before arrangements for monitoring their physical health in the community were agreed. Aware of the potential side effects of clozapine, some of the participants who started trial medication and experienced no side effects, judged that they were being given a placebo and withdrew from the study. Levels of adherence with pharmacological and other treatments have been reported to be low among people with borderline personality disorder.^39,46,47^ Adherence to clozapine is particularly important because people who miss more than 48 h of treatment need to be re-titrated and medication has to be stopped if people do not have regular blood monitoring. Problems with adherence are an important obstacle to the use of clozapine for people with borderline personality disorder.

While we were able to keep researchers masked to the allocation status of study participants, the high side-effect profile of clozapine, which includes sedation and hypersalivation, meant that many patients, researchers and clinicians believed they knew which arm of the trial they were in. Among 18 participants who guessed their allocation status after completing their 6-month follow-up assessment, 13 (72%) guessed correctly. Similarly, researchers correctly guessed the allocation status of 89% of study participants. These data suggest that most participants and researchers in placebo-controlled trials of clozapine are able to correctly guess their allocation status, threatening the internal validity of such trials.^48,49^

In addition to halting recruitment, the COVID-19 pandemic meant that researchers were no longer able to conduct face-to-face follow-up assessments. Instead, we arranged remote assessments by telephone or videoconferencing. Pairs of researchers completed all remote follow-up assessments, which helped ensure they were reliable. However, it was not possible to complete the Extrapyramidal Side Effects Scale as this requires a physical examination of the patient.^35^

The data from this trial do not form a sound basis for changing current clinical practice. Clinicians working on specialist inpatient units who have seen the mental health of people with borderline personality disorder improve with clozapine are likely to continue to prescribe it. The high cost of care and the relatively low cost of clozapine suggest that, if it was effective in reducing the amount of time people with BPD spend in hospital, clozapine would be a cost-effective treatment. But the logistical challenges we encountered on acute inpatient units when trying to ensure that clozapine was safely prescribed add to concerns about the use of clozapine for people with borderline personality disorder in this setting and in the community. The value placed on clozapine by clinicians working in specialist units is illustrated by the decision to start clozapine in two-thirds of the participants in the placebo arm of the trial who had been recruited in this setting. In contrast, none of the participants in the placebo arm who had been recruited from general adult mental health services were prescribed clozapine after the end of the study. Differences in the beliefs of clinicians working on specialist units and in general adult teams about the value of clozapine for people with severe BPD have implications for patient management. It is important to check that the general adult mental health team that will oversee the care of a person with severe BPD after they have been discharged from hospital is willing and able to provide ongoing physical health monitoring for people on clozapine before a decision is made to initiate this treatment in hospital.

Future studies of clozapine for inpatients with severe borderline personality disorder would need to identify units where clinicians supported the need for better information on the costs and benefits of this treatment. Consideration should be given to the use of an alternative antipsychotic medication, such as olanzapine, for those in the control arm of the trial, in order to make it less easy for study participants to correctly guess which arm of the trial they are in.

In conclusion, uncertainty remains about the role of clozapine in the treatment of inpatients with severe borderline personality disorder. However, this sense of uncertainty is not shared by many clinicians working on inpatient units in England and this may render randomised trials of clozapine unfeasible in this setting.
